# Neuropeptide Y neurons in the basolateral amygdala project to the nucleus accumbens and stimulate high-fat intake

**DOI:** 10.3389/fncel.2025.1565939

**Published:** 2025-03-27

**Authors:** Shunji Yamada, Kazunori Kojima, Masaki Tanaka

**Affiliations:** Department of Anatomy and Neurobiology, Graduate School of Medical Science, Kyoto Prefectural University of Medicine, Kyoto, Japan

**Keywords:** neuropeptide Y, basolateral amygdala (BLA), nucleus accumbens (NAc), high-fat diet, NPY receptor type 1

## Abstract

Neuropeptide Y (NPY) is a 36-amino acid neuropeptide that is widely expressed in the central nervous system, including in the nucleus accumbens (NAc), hypothalamus, and amygdala. The NAc involved in several behaviors, including reward, motivation processes, and feeding behavior. Here, we demonstrate in male mice that NPY input from the basolateral amygdala (BLA) to the NAc is involved in the preferential consumption of a high-fat diet (HFD). First, we demonstrated the NPY input to the NAc from the BLA by injecting adeno-associated virus (AAV)(retro)-FLEX-mCherry into the NAc of NPY-Cre mice. We also confirmed that BLA NPY neurons project exclusively to the NAc by injecting AAV(dj)-hSyn-FLEx -mGFP-2A-Synaptophysin-mRuby into the BLA. Usually, a HFD drives enhanced food intake than a standard chow diet after repetitive exposure. The optogenetic inactivation of BLA NPY neurons projecting to the NAc caused a significant decrease in HFD intake for a 1-h period, while optogenetic activation of these neurons induced the opposite effect. Furthermore, bilateral injection of an NPY receptor type 1 (Y1R) antagonist into the NAc significantly decreased HFD intake for 1-h period compared with vehicle injection, while, conversely, injection of a Y1R agonist enhanced HFD intake. These results suggest that BLA NPY neurons projecting to the NAc mediate preferential HFD intake via NAc-localized Y1R.

## 1 Introduction

Neuropeptide Y (NPY) is a 36-amino acid neuropeptide that was first described by Tatemoto et al. (1982). NPY-expressing neurons are widely distributed in the central nervous system, including in the nucleus accumbens (NAc), arcuate nucleus of the hypothalamus (ARH), and amygdala (Allen et al., 1983; Morris, 1989). NPY functions in various processes, including food intake, anxiety, memory, and processing of pain and itch (Tanaka et al., 2021). For example, injection of NPY into the paraventricular nucleus of the hypothalamus (PVN) elicits a strong feeding response (Stanley et al., 1985), and optogenetic activation of NPY/agouti related protein (AgRP)-expressing neurons in the ARH projecting to the PVN evokes feeding (Atasoy et al., 2012). These findings suggest that NPY neurons in the ARH mediate feeding behavior through the PVN. Additionally, using NPY-Cre mice and chemogenetic strategies, we have reported that NPY neurons in the NAc are involved in anxiety behavior (Yamada et al., 2020) and that they project to the lateral hypothalamus (LH) (Yamada et al., 2021).

Several lines of evidence indicate that NPY administration into the NAc can mediate reward behaviors. Bilateral injection of NPY into the NAc can cause reward-related behavior in the conditioned place preference test (Brown et al., 2000). Ethanol self-administration increases dose-dependently after administration of NPY or a NPY receptor type I (Y1R) agonist into the NAc (Borkar et al., 2016). In rats on the free-choice, high-fat, high-sugar diet, injection of NPY into the NAc increases the intake of fat, but not sugar, via Y1R (van den Heuvel et al., 2015). NPY in the rat NAc has been thought to originate from local interneurons (Kawaguchi et al., 1995) and/or from neurons projecting from the ARH (van den Heuvel et al., 2015). However, in mice, the origins of NPY inputs to the NAc remain to be clarified.

The NAc receives afferents from various brain regions, including the thalamus, medial prefrontal cortex, and basolateral amygdala (BLA) (Brog et al., 1993). Projection neurons from the BLA to the NAc are thought to modulate reward-seeking behavior (Cador et al., 1989; Di Ciano and Everitt, 2004; Ambroggi et al., 2008) and NPY-expressing neurons exist in the BLA (Morris, 1989). The BLA is therefore a candidate for the origin of the NPY inputs to the NAc that are associated with reward. Here, we used NPY-Cre mice to examine the source of NPY fibers in the NAc and whether these NPY inputs are involved in the reward behavior of palatable food consumption. We confirmed that BLA NPY neurons project directly to the NAc and demonstrated that optogenetic manipulations of NPY terminals from the BLA alter high-fat diet (HFD) intake.

## 2 Materials and methods

### 2.1 Animals

B6.FVB(Cg)-Tg(NPY-cre)RH26Gsat/Mmucd (037423-UCD, NPY-Cre) sperm were purchased from the Mutant Mouse Resource and Research Center (Auckland, CA, USA) and used by the RIKEN BioResource Research Center (Ibaraki, Japan) to generate NPY-Cre mice. Previously, we used the NPY-Cre mice and validated specificity of Cre expression in the NPY neurons (Yamada et al., 2020, 2021). In the present study, male mice (6–10 weeks of age) were used. The mice were kept under a 12-h light/dark cycle (lights on at 8 a.m.). Standard food pellets and water were provided *ad libitum*. All animal experimental procedures, including production and maintenance protocols, and behavioral studies, were reviewed and approved by the Animal Care and Use Committee of the Kyoto Prefectural University of Medicine (M2021-180&182, M2022-159&161, M2023-157&159).

### 2.2 High-fat diet (HFD) intake test

An HFD intake test was performed with reference to previously described studies (Stanley et al., 1985; Christoffel et al., 2021). One week after the most recent surgery, mice were given daily access to a high-fat pellet (20% carbohydrate, 20% protein, 60% fat; #D12492, Research Diets Inc., New Brunswick, NJ, USA) for 1 h at the same time each day. The HFD intake during this hour was measured every day for 10 days. After HFD exposure for 10 days, levels of HFD intake in mice had become stable. For optogenetic or pharmacologic behavioral experiments, mice treated with HFD for 10 days were habituated to the experimental cage for 3 days before the first day of light stimulation or drug/vehicle injection. On the experiment day, mice were exposed to HFD for 1 h at 11:00 - 16:00 (light cycle) and the intake was measured.

### 2.3 Adeno-associated virus (AAV) preparation

pAAV-EF1a-DIO-mCherry (Addgene plasmid #50462; RRID:Addgene_50462)^1^ was a gift from Bryan Roth. rAAV2-retro helper (Addgene plasmid #81070; RRID:Addgene_81070)^2^ was a gift from Alla Karpova and David Schaffer (Tervo et al., 2016). pAAV-hSyn-FLEx-mGFP-2A-Synaptophysin-mRuby (Addgene plasmid #71760; RRID:Addgene_71760)^3^ was a gift from Liqun Luo (Beier et al., 2015). pAAV-EF1a-double floxed-hChR2(H134R)-EYFP-WPRE-HGHpA (Addgene plasmid #20298; RRID:Addgene_20298)^4^ and pAAV-Ef1a-DIO -eNpHR3.0-EYFP (Addgene plasmid #26966; RRID:Addgene_26966)^5^ were gifts from Karl Deisseroth (Gradinaru et al., 2010). For recombinant AAV (rAAV) production, HEK293 cells were cotransfected with the vectors pAAV-DJ (Cell Biolabs, San Diego, CA, USA) or rAAV2-retro helper and pHelper (Takara Bio Inc., Shiga, Japan) using the AAVpro Helper Free system (Takara Bio Inc.). rAAV particles were extracted and purified using the AAVpro Purification Kit (Takara Bio Inc.), according to the manufacturer’s instructions. rAAV titers were determined by quantitative PCR using the AAVpro Titration Kit (Takara Bio Inc.). Aliquoted rAAVs were stored at −80°C.

### 2.4 Stereotaxic virus injections

Mice were anesthetized with a subcutaneous injection of a midazolam/medetomidine/butorphanol cocktail (4, 0.3, and 5 mg/kg, respectively) and placed on a stereotaxic apparatus (Narishige, Tokyo, Japan). AAVs were microinjected (0.2–1 μl/site) into the NAc [anteriorposterior (AP), +1.2 mm from the bregma; mediolateral (ML), +0.8 mm from the midline; dorsoventral (DV), 4.3 mm below the skull surface] or BLA (AP, −1.5 mm from the bregma; ML, +3.3 mm from the midline; DV, 4.7 mm below the skull surface) using a 30-gauge Hamilton syringe needle (Hamilton, Reno, NV, USA) at a rate of 0.1 μl/min. The needle was kept in place for 5–10 min and then the needle was slowly removed.

After surgery, anesthesia was immediately reversed with a subcutaneous injection of atipamezole, and mice were housed individually for recovery. Viral injection, optic fiber placement, and cannula implantation were verified by fluorescence imaging or tip location. Mice in which injected virus, implanted optic fibers, or cannulas were not correctly targeted were excluded from the analysis. Cre-dependent expression in NPY-Cre mice using AAVs were confirmed in our previous report (Yamada et al., 2020).

### 2.5 Tracing experiments

For retrograde labeling of NPY neurons projecting to the NAc, the NAc of NPY-Cre mice was unilaterally injected with 0.2 μl of AAV(retro)-FLEX-mCherry (0.7 × 10^12^ viral genome particles (vg)/mL) (*n* = 3). For anterograde labeling of NPY neurons in the BLA, the BLA of NPY-Cre mice was unilaterally injected with 0.2 μl of AAV(dj)-FLEX-mGFP-t2A-SynRuby (0.2 × 10^11^ vg/mL) (*n* = 3). mCherry and mGFP expression were allowed to develop for more than 2 weeks before the mice were deeply anesthetized and perfused. We previously confirmed that 0.2 μl of AAV injection into the NAc causes Cre-dependent expression only in the NAc but not BLA through diffusion (Yamada et al., 2020, 2021).

### 2.6 Optogenetic experiments and HFD intake test

The BLA of NPY-Cre mice was bilaterally injected with 0.8 μl of AAV(dj)-DIO-eNpHR3.0-EYFP (0.2 × 10^12^ vg/mL), 0.5 μl of AAV(dj)-DIO- hChR2(H134R)-EYFP-WPRE-HGHpA (0.5 × 10^12^ vg/mL) or 0.5 μl of AAV(dj)-DIO-GFP (0.1 × 10^12^ vg/mL) (*n* = 8 for each construct). One week after virus injection, optic fibers attached to a ferrule (BrainScience Idea. Co., Ltd, Osaka, Japan) were implanted bilaterally above the NAc (angle 10°, AP, + 1.5 mm from the bregma; ML, + 1.6 mm from the midline; DV, 4.7 mm below the skull surface) and fixed to the skull with two anchor screws and dental cement (Shofu Inc., Kyoto, Japan). Each optic fiber was 200 μm in diameter and had a 0.39 numerical aperture. The ferrule was 1.25 mm in diameter and made from zirconia. On the experimental day, the ferrules were connected to a laser output device (TESLIGHT; BrainScience Idea) through optic patch cords (Thorlabs, Newton, NJ, USA). The mice were habituated to the attached patch cords prior to the test start for at least 1 h. The mice then received either blue light (473 nm, 20 Hz, 5 ms pulse) or green light (530 nm, continuously) stimulation for 5 min prior to the start of the 1-h HFD intake test (light-on). As a control experiment, the 1-h HFD test was performed on a separate day using the same mice with patch cords but no light stimulation (light-off). For some mice, the 1-h HFD intake test was first performed after light-on treatment and subsequently after light-off treatment. For other mice, the light-on/off order was reversed.

### 2.7 Real-time RT-PCR for NPY receptor type 1 (Y1R), type 2 (Y2R), type 5 (Y5R), and NPY

Mice fed a HFD for 10 days (HFD-treated mice) or non-HFD fed (non-HFD mice) mice were decapitated between 15:00 and 17:00 h. After removing the brain, 2 mm-thick coronal sections at the level of the NAc were prepared according to the brain atlas (Franklin and Paxinos, 2007). The bilateral NAc was then punched out using an 18-gauge stainless-steel tube. Total RNA was extracted using Sepasol-RNA I super G (Nacalai tesque, Kyoto, Japan). Complementary DNA (cDNA) was synthesized from 0.5 μg of each total RNA sample using ReverTra Ace PCR RT Master Mix (Toyobo, Osaka, Japan).

The Lightcycler 480 sequencer (Roche Diagnostics, Indianapolis, IN, USA) was used for real-time RT-PCR assays. Primer sets for Y1R, Y2R, Y5R, NPY, and glyceraldehyde-3-phosphate dehydrogenase (GAPDH) as the internal control were purchased from Perfect Real time support system (Takara Bio Inc.). For each reaction, each primer set was mixed with 5 μl cDNA and Lightcycler 480 SYBR Green I Master (Roche Diagnostics). Relative fold-changes in gene expression, normalized against GAPDH, were calculated using the comparative Cp (threshold cycle number) method.

### 2.8 NPY Y1 receptor antagonist or agonist injection into the NAc and HFD intake test

For pharmacological experiments, a 26-gauge stainless-steel double guide cannula (C235GS-5-2.9/SPC; Plastics One, Roanoke, VA, USA) was implanted bilaterally above the NAc (AP, +1.2 mm from the bregma; ML, ±1.0 mm from the midline; DV, 4.3 mm below the skull surface) according to the mouse brain atlas (Franklin and Paxinos, 2007) and fixed the skull with two anchor screws and dental cement (Shofu Inc.). On the experiment day, BIBO 3304 trifluorooacetate (a Y1R antagonist; Selleck, Houston, TX, USA) was dissolved to 4 mM in saline containing 4% DMSO and [Leu^31^, Pro^34^]-NPY (a Y1R agonist; Peptide Institute, Osaka, Japan) was dissolved to 0.2 mM in saline immediately before injection. Y1R antagonist and agonist doses were chosen with reference to previous studies (Wieland et al., 1998; Smith et al., 2022). Freely-moving mice received a single infusion of these drugs (*n* = 12 for the Y1R antagonist; *n* = 8 for the Y1R agonist) into the NAc at the rate of 0.1 μl/min for 5 min using a microsyringe pump (BrainScience Idea) through a 33-gauge double internal cannula (C235IS-5-2.0/SPC; Plastics One). The cannula was then removed, and the 1-h HFD intake test was performed 5 min later. As a control, the same mice received vehicle (saline containing 4% DMSO or saline) into the NAc in the same manner and the 1-h HFD intake test was then performed. The interval between HFD intake tests after drug or vehicle injection was 4–6 days. The order of antagonist/agonist or vehicle injection is random.

### 2.9 Immunohistochemistry

Mice were anesthetized with intraperitoneal pentobarbital (Somnopentyl; Kyouritsu Seiyaku, Tokyo, Japan) and perfused with physiological saline followed by 4% paraformaldehyde in 0.05 M phosphate buffer. For mCherry and NPY double immunohistochemistry, AAV(retro)-FLEX-mCherry injected-mice were injected 75 μg colchicine (dissolve in saline) into the lateral ventricle 24 h before perfusion to visualize cell bodies of NPY-positive neurons in the BLA. To confirm the effect of optogenetic activation on neurons in the NAc, AAV(dj)-FLEX-ChR2-EYFP-injected mice (*n* = 3) were unilaterally stimulated by blue light for 1 h before perfusion. To confirm the effect of optogenetic inhibition on neurons in the NAc, AAV(dj)-FLEX-eNpHR3-EYFP-injected (*n* = 3) and AAV(dj)-FLEX-GFP-injected mice (*n* = 3) were given HFD and applied green light for 1 h before perfusion. The brain was immediately removed, post-fixed in the same fixative overnight at 4°C, and then dehydrated in 30% sucrose in 0.05 M phosphate buffer at 4°C for 4–6 days. Serial coronal sections (40 μm thick) were prepared using a cryostat (CM 3050 S; Leica, Wetzlar, Germany).

For retrograde tracing of NPY neurons projecting to the NAc, we performed mCherry immunohistochemistry. Every fourth section was incubated with 0.3% H_2_O_2_ and 0.3% Triton X-100 in PBS for 30 min, and then in PBS containing 2% normal goat serum (NGS) and 0.1% Triton X-100 for 1 h at room temperature (RT). Sections were then incubated with primary rabbit antiserum against mCherry (1:2,000; ab167453, Abcam, Cambridge, UK) for 72 h at 4°C. Immunoreactive neurons were visualized using a streptavidin-biotin kit (Nichirei, Tokyo, Japan), followed by 3,3′-diaminobenzidine (DAB), as we have described previously (Takanami et al., 2010; Yamada and Kawata, 2014).

For mGFP, c-Fos or mCherry and NPY double fluorescence immunohistochemistry, every fourth section was incubated with 2% NGS in PBS for 1 h, and then with primary antibody for 72 h at 4°C. After washing with PBS, the sections were incubated with secondary antibody. Concentrations and sources for antibodies were as follows: rabbit antiserum against GFP (1:2,000; ab6556, Abcam), rabbit antiserum against c-Fos (1:2000; ab190289, Abcam), rabbit antiserum against NPY (1:2,000; D7Y5A, Cell Signaling Technology, Danvers, USA), rat antiserum against mCherry (1:2,000; M11217, Thermo Fisher Scientific), Alexa Fluor 488-labeled donkey anti-rabbit IgG (1:1,000; A21206, Thermo Fisher Scientific, Waltham, MA, USA), Alexa Fluor 555-labeled donkey anti-rabbit IgG (1:1,000; A31572, Thermo Fisher), and Alexa Fluor 555-labeled donkey anti-rat IgG (1:1,000; ab150154, Abcam).

Sections reacted with DAB were observed under a light microscope (BX50; Olympus, Tokyo, Japan) and images were captured using a CCD camera (DP 21; Olympus). High or low magnification fluorescence images were obtained using a LSM510META confocal laser-scanning microscope (Carl Zeiss, Jena, Germany) or a BioZero BZ-X 710 Fluorescence digital microscope (Keyence, Osaka, Japan), respectively.

### 2.10 Statistical analysis

All values are expressed as the mean ± SEM. Significant differences in 1-h HFD intake between light-on and light-off in ChR2-, eNpHR-, and GFP-mice and between antagonist or agonist and vehicle-injected mice were evaluated by the two-tailed paired *t*-test.

## 3 Results

### 3.1 Retrograde tracing of NPY fibers in the NAc

**FIGURE 1:**
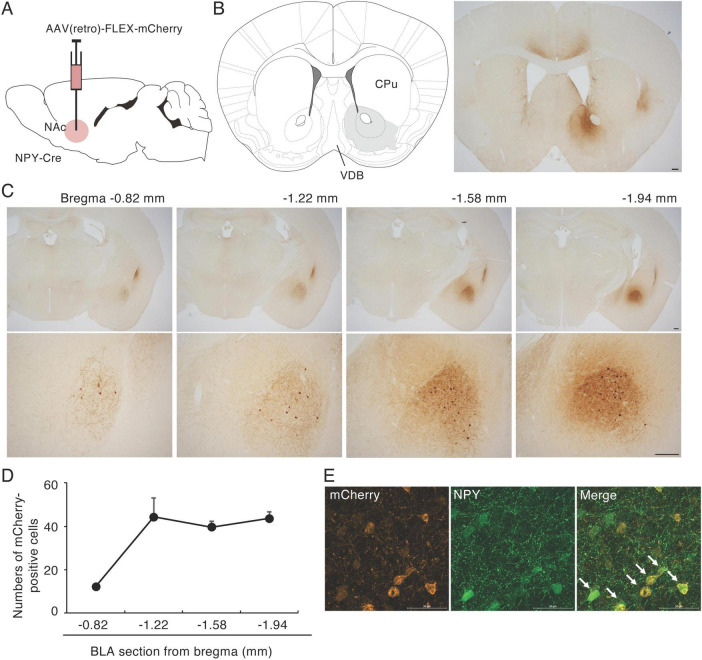
Retrograde tracing of NPY fibers in the NAc. **(A)** Schematic image of the experimental design for retrograde tracing. **(B)** Coronal section from the mouse brain atlas showing the position of the NAc (left) and representative photograph showing the site of mCherry expression (brown) at the injection site of AAV(retro)-FLEX-mCherry into the NAc (right). **(C)** mCherry-positive cell bodies were specifically located in the CLA and the BLA. Numbers are the mean distance to the bregma (upper). The BLA regions in the upper images are magnified in the lower images. **(D)** The distribution of mCherry-positive cells through the BLA. Values are means ± SEM (*n* = 3). The BLA was divided into the four sections according to the brain atlas (Franklin and Paxinos, 2007). **(E)** In AAV(retro)-FLEX-mCherry-NAc-injected mice, most mCherry-positive cells (red) in the BLA were NPY (green) positive (arrows). Scale bars = 0.2 mm in panels **(B,C)**, 0.05 mm in panel **(E)**.

To investigate the origin(s) of NPY fibers in the NAc, we injected AAV(retro)-FLEX-mCherry, which permits retrograde expression of mCherry in projection neurons, into the NAc of NPY-Cre mice (Figures 1A, B and Supplementary Figure 1). We have confirmed that the AAV administered to the NAc does not spread much to the substantia innominate (SI) and ventral pallidal (VP) portions of the basal forebrain (SI/VP) where is a projection site of NPY-expressing GABAergic neurons in the BLA (McDonald et al., 2012; Supplementary Figure 1B). We analyzed the localization of mCherry-positive cell bodies in coronal sections spanning the entire brain and found many mCherry-positive cell bodies in the BLA (Figure 1C) and a few in the claustrum (CLA; Supplementary Figure 2). Dendrites of retrogradely labeled mCherry-positive neurons in the BLA bore spines (Supplementary Figure 1C), indicating that a part of NPY neurons in the BLA are principal neurons projecting to the NAc. We did not detect clusters of mCherry-positive neurons in other brain regions. The mCherry-positive cell bodies were spread throughout the BLA, with a longitudinal distance from 0.82 mm to 1.94 mm posterior to the bregma, according to the brain atlas (Franklin and Paxinos, 2007). mCherry-positive cells were more numerous in the posterior BLA compared with the anterior BLA (Figure 1D). Using mCherry and NPY double immunohistochemistry, we confirmed that 80.8% of mCherry-positive neurons in the BLA were NPY positive and 98% of NPY-positive neurons in the BLA were mCherry positive (Figure 1E). mCherry-positive cell bodies were also spread throughout the CLA, with a longitudinal distance from 1.18 mm anterior to 1.58 mm posterior to the bregma (Supplementary Figure 2). However, we did not observe any mCherry-positive cell bodies in the ARH where many NPY-positive cells were located (Figure 1 and Supplementary Figure 2).

### 3.2 Anterograde tracing of NPY neurons in the BLA

**FIGURE 2:**
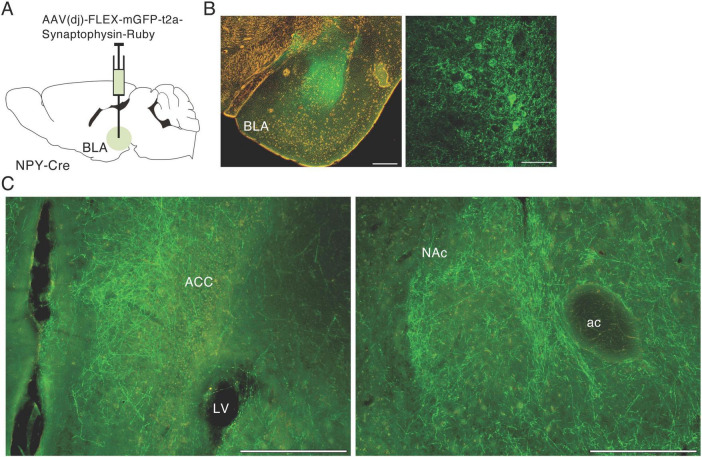
Anterograde tracing of NPY neurons in the BLA. **(A)** Schematic image of the experimental design for anterograde tracing. **(B)** Representative photograph showing GFP expression (green) at the injection site in AAV(DJ)-FLEX-mGFP-SynRnby-BLA-injected mice (left); the right-hand photograph is a magnified image of the BLA. **(C)** Photographs of GFP-positive fibers from the BLA in the anterior cingulate cortex (ACC, left) and NAc (right). ac, anterior commissure; LV, lateral ventricle. Scale bars = 0.2 mm.

To confirm the finding of a NPY projection from the BLA to NAc, we injected AAV(dj)-FLEX-mGFP-t2A-synaptophysin-mRuby into the BLA of NPY-Cre mice (Figure 2A). mGFP is a palmitoylated GFP that accumulates in the cell membrane and can be used to clearly visualize neural fibers of BLA NPY neurons. Many mGFP-positive cell bodies were detected in the BLA around the injection site (Figure 2B). We also observed mGFP-positive fibers in the anterior cingulate cortex (ACC) and the medial part of the NAc, including in the shell and core regions (Figure 2C). These results indicate that a proportion of NPY neurons in the BLA project to the medial region of the NAc in mice.

### 3.3 Inactivation of BLA-NAc NPY neurons decreases HFD intake

**FIGURE 3:**
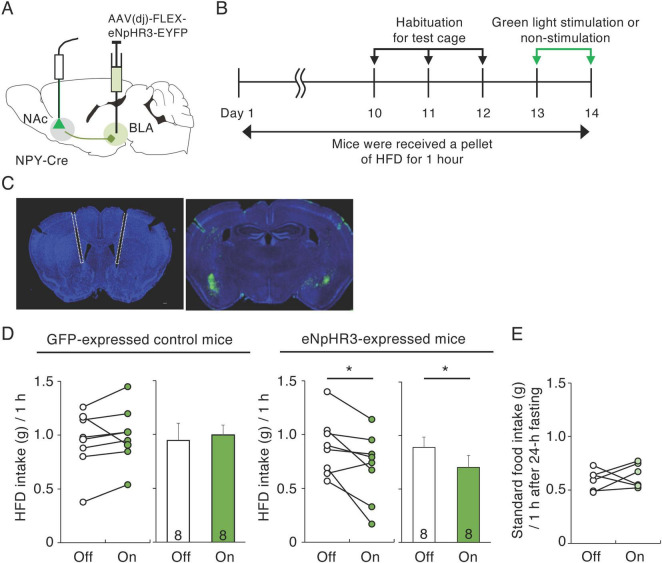
Inactivation of BLA-NAc NPY neurons decreases HFD intake. **(A)** Schematic image of the experimental design for inactivation of BLA-NAc NPY neurons. **(B)** Schematic depiction of the experimental timeline. **(C)** Representative photographs of light fiber placement above the NAc (white dotted lines) (left) and eNpHR3-EYFP (green) infection in the BLA (right). **(D)** 1-h HFD intake during green light-off (white) or -on (green) in GFP-expressing control and eNpHR3-expressing mice. Left panels illustrate individual subjects and right panels show the means ± SEM. **(E)** 1-h standard food intake 24 h after fasting during green light-off (white) or -on (light green) in eNpHR3-expressing mice. Statistical differences between light-off and light-on were determined by paired *t*-test (*P = 0.033).

Injection of NPY into the NAc can enhance HFD intake in rats (van den Heuvel et al., 2015). Meanwhile, the BLA mediates aversive and appetitive stimuli (Zhang et al., 2021), and BLA neurons projecting to the NAc mediate reward-seeking behavior (Zhou et al., 2022). We therefore hypothesized that NPY neurons in the BLA that project to the NAc are involved in palatable food consumption. To investigate the regulation of palatable food intake by BLA NPY neurons projecting to the NAc, we employed a “daily 1-h access HFD model” modified from a previous report (Christoffel et al., 2021). In this model, mice are at liberty to consume a HFD for 1 h during the light cycle. To inactivate NPY inputs to the NAc from the BLA, we injected AAV(dj)-DIO-eNpHR3.0-EYFP or AAV-DIO-GFP, as a control, into the BLA of NPY-Cre mice and delivered 530 nm green light through optical fibers implanted into the medial part of the NAc throughout the 1-h HFD test (Figures 3A–C). We investigated the effects of inactivating BLA-NAc NPY neurons on HFD intake for 1 h 10 days after habituation to HFD intake (Figure 3B). The habituation period resulted in relatively stable HFD intake. In the last day of habituation period, mice consumed 1.02 ± 0.06 g HFD during 1 h (*n* = 23). In the control mice expressing GFP, application of green light did not affect the 1-h HFD intake (Figure 3D). However, application of green light to the eNpHR-expressed mice caused a significant decrease (*n* = 8, *p* = 0.033, paired *t*-test) in 1-h HFD intake compared with that in the same mice not exposed to green light (Figure 3D). Next, to confirm whether suppression of HFD intake in response to green light was induced by inhibition of appetite or reward motivation, we investigated the effect of inactivating BLA-NAc NPY neurons on standard food (not high-fat) intake after fasting for 24-h. We found that BLA-NAc NPY neuron inactivation did not affect standard food intake after fasting (Figure 3E). We confirmed projection of eNpHR3-EYFP-expressing fibers in the NAc (Supplementary Figure 3A) and response of neurons in the NAc after optogenetic inhibition of BLA-NAc NPY neurons using c-Fos immunohistochemistry. Previous report suggested that the exposure of HFD induces an increase in c-Fos expression in the NAc (Valdivia et al., 2014). We showed that the number of c-Fos-expressing cells in the NAc after HFD exposure with green light application was significantly lower in the eNpHR3-expressing mice than that in GFP-expressing control mice (*n* = 3, *p* = 0.005, Student’s *t*-test) (Supplementary Figures 3B, C).

### 3.4 Activation of BLA-NAc NPY neurons increases HFD intake

**FIGURE 4:**
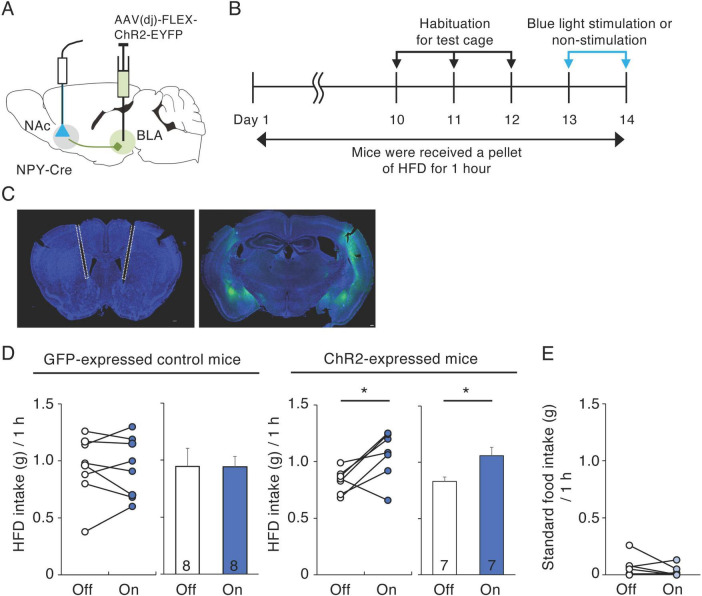
Activation of BLA-NAc NPY neurons increases HFD intake. **(A)** Schematic image of the experimental design for activation of BLA-NAc NPY neurons. **(B)** Schematic depiction of the experimental timeline. **(C)** Representative photographs of light fiber placement above the NAc (white dotted lines) (left) and ChR2-EYFP (green) infection in the BLA (right). **(D)** 1-h HFD intake during blue light-off (white) or -on (blue) in GFP-expressing control and ChR2-expressing mice. Left panels illustrate individual subjects and right panels show the means ± SEM. **(E)** 1-h standard food intake during blue light-off (white) or -on (light blue) in ChR2-expressing mice. Statistical differences between light-off and light-on were determined by paired *t*-test (*P = 0.032).

To activate NPY inputs to the NAc from the BLA, we injected AAV(dj)-DIO-ChR2-EYFP or AAV-DIO-GFP into the BLA of NPY-Cre mice and exposed the mice to 474 nm blue light at 20 Hz through optical fibers implanted into the medial part of the NAc throughout the 1-h HFD test (Figures 4A–C). We investigated the effects of activating BLA-NAc NPY neurons on HFD intake during 1 h, 10 days after habituation to HFD intake (Figure 4B). In the GFP-expressed control mice, application of blue light did not affect 1-h HFD intake (Figure 4D). However, application of blue light to the ChR2-expressed mice caused a significant increase in 1-h HFD intake compared with that in the same mice not exposed to blue light (*n* = 7, *p* = 0.032, paired *t*-test) (Figure 4D). Next, we investigated the effect of activating BLA-NAc NPY neurons on standard food intake. We found that activation of BLA-NAc NPY neurons did not influence standard food intake (Figure 4E), indicating that activation of BLA-NAc NPY neurons enhances motivation to ingest HFD but not standard food. We confirmed projection of ChR2-EYFP-expressing fibers in the NAc (Supplementary Figure 3D) and response of neurons in the NAc after optogenetic stimulation of BLA-NAc NPY neurons using c-Fos immunohistochemistry. We showed that the number of c-Fos-expressing cells in the NAc tend to be higher in BLA-NAc NPY neurons-stimulated side than that in no-stimulated side (*n* = 3, *p* = 0.062, Student’s *t*-test) (Supplementary Figures 3E, F).

### 3.5 Effects of Y1R antagonist and agonist injection into the NAc on HFD intake

**FIGURE 5:**
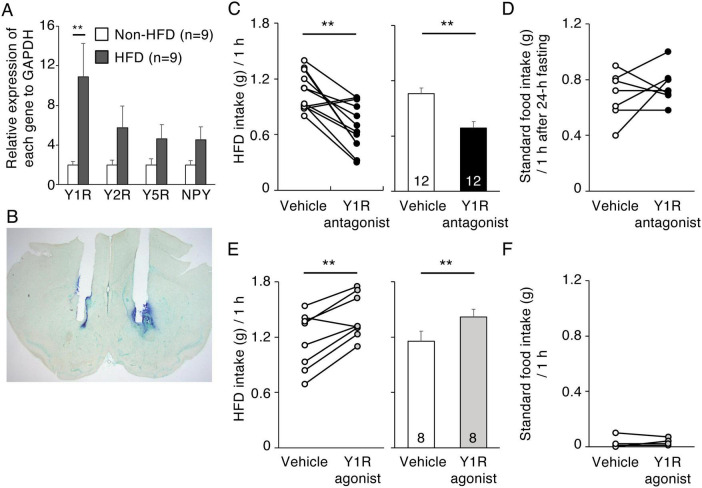
Effects of Y1R antagonist and agonist injection into the NAc on HFD intake. **(A)** The effect of HFD intake on Y1R, Y2R, Y5R, and NPY mRNA levels in the NAc. Y1R, Y2R, Y5R, and NPY mRNA levels in the NAc in non-HFD-treated (white bars) and HFD-treated (black bars) mice. Values are means ± SEM. Data were analyzed by the Student’s *t*-test. Y1R mRNA levels in HFD-treated mice were significantly (P** = 0.0023, Student’s *t*-test) higher than in non-HFD-treated mice. **(B)** Representative photograph of the double cannula implantation into the NAc. **(C,D)** 1-h HFD intake **(C)** and 1-h standard food intake after fasting for 24-h **(D)** 10 min after vehicle (white) or Y1R antagonist (black) injection into the NAc. **(E,F)** 1-h HFD intake **(E)** and 1-h standard food intake **(F)** 10 min after vehicle (white) or Y1R agonist (gray) injection into the NAc. Left panels illustrate individual subjects and right panels show means ± SEM for 1-h HFD intake **(C,E)**.

Five NPY receptors have been identified in mammals, Y1R, Y2R, Y4R, Y5R, and y6R (Michel et al., 1998). Y1R and Y2R are abundant in the NAc of rats (Kask et al., 2002) and NPY-induced HFD intake can be inhibited in rats by pretreatment with a Y1R antagonist (van den Heuvel et al., 2015). Our quantitative RT-PCR analysis showed the expression level of Y1R in the NAc of HFD-treated mice to be significantly higher than that in non-HFD control mice (*n* = 10, *p* = 0.023, Student’s *t*-test). However, the expression levels of Y2R, Y5R, and NPY were not different between HFD-treated and control mice (Figure 5A). We then examined whether pharmacological suppression or activation of Y1R in the NAc influenced 1-h HFD intake in mice. Photomicrographs of cannula placements into the NAc in representative animals are shown in Figure 5B. Histological examination revealed that Y1R antagonist (triangles) and agonist (circles) were accurately injected into the NAc (Supplementary Figure 4). In Y1R antagonist-injected mice, 1-h HFD intake was significantly lower than that in vehicle-injected mice (*n* = 12, *p* = 0.001, paired *t*-test) (Figure 5C). In contrast, Y1R antagonist injection did not influence in 1-h standard food intake after 24-h fasting (Figure 5D). In addition, 1-h HFD intake by Y1R agonist-injected mice was significantly higher than that by vehicle-injected mice (*n* = 8, *p* = 0.003, paired *t*-test) (Figure 5E) but Y1R agonist injection did not influence in 1-h standard food intake (Figure 5F).

## 4 Discussion

Pharmacological studies involving injection of NPY or NPY receptor agonists/antagonists into the NAc have demonstrated that NPY in the NAc affects alcohol intake, drug addiction, food intake, anxiety, and depression (Tanaka et al., 2021). However, these methods are limited for investigating the involvement of NPY-related neural circuits in such functions. The Cre/loxP recombination system in transgenic mice can be used for neuron-specific manipulation in functional and anatomical studies. Using NPY-Cre mice, we previously showed that NPY neurons in the NAc project to the LH and are involved in anxiety behavior (Yamada et al., 2020, 2021). In the present study, we demonstrated that the NAc receives NPY-positive neural inputs from the BLA and that these afferents affect HFD intake.

Previous studies showed that many BLA neurons project to the NAc (Huang et al., 2021; Zhou et al., 2022) and there are some NPY-expressing principal neurons in the BLA (Vereczki et al., 2021). To our knowledge, we are the first to report that a portion of BLA neurons innervating the NAc express NPY. Since NPY-expressing GABAergic neurons project to the SI/VP where is adjacent to the NAc (McDonald et al., 2012), BLA NPY neurons project to the NAc may be GABAergic. The involvement of BLA neurons that project to the NAc in reward is controversial. For example, optogenetic activation of NAc-projecting BLA neurons promoted reward-seeking behavior (Zhou et al., 2022). Zhang et al. (2021) showed that BLA neurons that project to the NAc are required for negative behavior, such as the learning and expression of punishment-avoidance, but not for reward-seeking behavior. This discrepancy may result from the differential activation of subsets of BLA neurons that project to the NAc that have different characteristics. Here, we demonstrated that specific activation of NPY-expressing BLA neurons projecting to the NAc mediate binge HFD consumption. Because the mice ate only HFD, but not standard food, during the 1-h HFD test, HFD is considered palatable and also reward for mice. Eating palatable food regardless of energy states is hedonic (Halpern et al., 2013; Christoffel et al., 2021), therefore, NPY originating from BLA neurons that project to the NAc, is likely to contribute to hedonic eating in male mice.

Migita et al. (2001) detected Y1R immunoreactivity in the rat NAc, although its localization was not defined to cell bodies or fibers. Y1R mRNA has been localized to rat NAc cells by radioisotopic *in situ* hybridization (Kishi et al., 2005). In addition, Smith et al. (2022) showed that a Y1R agonist modulates synaptic transmission in mouse NAc neurons using whole-cell patch-clamp electrophysiology. In this study, we confirmed Y1R mRNA expression in the NAc using real-time PCR and demonstrated a pharmacological effect of Y1R antagonist/agonist injection into the NAc on HFD intake. These results also indicate the existence of Y1R-expressing cells in the NAc of mice. Enkephalin-expressing neurons express Y1R in the NAc of rats (van den Heuvel et al., 2015) and enkephalin agonists increase the intake of high-fat and/or sweet diets (Levine and Billington, 2004); therefore, BLA NPY neurons that project to the NAc might enhance HFD intake through these Y1R-expressing enkephalin neurons. However, NPY receptors are also found on excitatory and monoaminergic terminals in the NAc (Smith et al., 2022). Therefore, it is also conceivable that these BLA neurons that project to the NAc may enhance HFD intake by modulating glutamate and monoamine transmission. Our study has certain limitations. Due to technical and instrumental challenges, we did not directly prove whether NPY-expressing neurons in the BLA release NPY and whether this release is involved in HFD intake. Further studies, such as combining chemogenetic approach with pharmacological analyses and/or detecting endogenous NPY using a GRAB sensor, are needed to identify the involvement of NPY.

In the present study, optogenetic activation of BLA-NAc NPY neurons did not affect standard food intake for 1 h during the light cycle or after fasting for 24-h. Previous studies have also reported that bilateral NPY injection into the NAc does not increase intake of standard food (Brown et al., 2000) and that fasting and refeeding do not change NPY concentrations in the NAc (Beck et al., 1990). It is well-established that ARH AgRP/NPY neurons are important in homeostatic food intake (Atasoy et al., 2012) and that NPY concentration in the ARH is altered by fasting and refeeding (Beck et al., 1990). Taking these findings together, we suggest that BLA-NAc NPY neurons are not involved in homeostatic food intake but are involved in the intake of palatable food, such as HFD.

Retrograde tracing and NPY immunohistochemistry have indicated that NPY neurons in the rat ARH project to the NAc (van den Heuvel et al., 2015). However, in mice, AgRP-immunoreactive fibers were not detected in the NAc, cerebral cortex, hippocampus, or caudate putamen (Broberger et al., 1998). AgRP mRNA-expressing cells are only located in the ARH and 95% of these cells co-express NPY mRNA. Furthermore, in mice, these NPY/AgRP neurons in the ARH do not send axons to the NAc (Broberger et al., 1998). These findings are consistent with our observation of no mCherry-positive cell bodies in the mouse ARH after injection of AAV (retro)-FLEX-mCherry into the NAc. In mice, there may be different roles for NPY neurons in the ARH and BLA regarding food intake; one is dependent on energy status and the other is not.

We also detected CLA neurons that project to the NAc. NPY-immunoreactive neurons in the CLA are present in rats (Allen et al., 1983; Lipiec-Borowicz et al., 2024). Niu et al. (2022) suggested that the CLA mediates the stress-induced anxiety response in mice. NPY injection into the lateral ventricle, near the NAc, causes anxiolytic-like effects in rats (Broqua et al., 1995; Heilig, 2004), therefore, NPY input from the CLA into the NAc may modulate stress and anxiety behaviors. In addition, we also found that NPY neurons in the BLA project to the ACC. Although there are Y1R mRNA signals in the ACC of mice (Kishi et al., 2005), function of NPY in the ACC has not been understood yet. Additional studies are needed to investigate CLA-NAc and BLA-ACC NPY neurons.

The present study demonstrated direct NPY input from the BLA to the NAc and that these NPY afferents enhanced the 1-h HFD intake of male mice. Injection of Y1R antagonist or agonist caused a decrease or increase in 1-h HFD intake respectively, suggesting that BLA NPY neurons regulate palatable food consumption via the Y1R-expressed cells in the NAc.

## Data Availability

The original contributions presented in this study are included in this article/Supplementary material, further inquiries can be directed to the corresponding author.

## Footnotes

1
http://www.addgene.org/50462/

2
http://www.addgene.org/81070/

3
http://www.addgene.org/71760/

4
http://www.addgene.org/20298/

5
http://www.addgene.org/26966/

## Supplementary material

Supplementary Figure 1 mCherry-labeled fibers in the NAc and BLA. **(A,B)** Representative photograph showing the mCherry expression (brown) in the NAc **(A)** and substantia innominate (SI) and ventral pallidal portions of the basal forebrain (SI/VP) **(B)** in the AAV(retro)-FLEX-mCherry-injected mice into the NAc. **(C)** Representative photograph showing mCherry-positive cell and fibers in the BLA. Arrows indicate mCherry-positive dendrites bearing spines. ac; anterior commissure, CPu, caudate putamen. Scale bars = 0.1 mm.

Supplementary Figure 2 Retrograde tracing of NPY fibers in the NAc. **(A)** Representative photographs of horizontal brain sections of AAV(retro)-FLEX-mCherry-NAc-injected mice at the level of the CLA (left) and NAc/BLA (right). **(B)** Representative coronal section showing mCherry-positive cell bodies in the CLA. Numbers indicate distance to the bregma. **(C)** Representative photograph of the ARH in AAV(retro)-FLEX-mCherry-NAc-injected mice. There are no mCherry-positive cells in the ARH. Scale bars = 0.2 mm.

Supplementary Figure 3 Response of neurons in the NAc after optogenetic inhibition and stimulation of BLA-NAc NPY neurons. **(A)** Representative photograph of eNpHR3-EYFP-expressing fibers in the NAc. **(B)** Representative photograph of c-Fos expressing cells (red) in the NAc after HFD exposure with green light application for 1 h in GFP-expressing control (left) and eNpHR3-expressing mice (right). **(C)** The number of c-Fos expressing cells in the NAc was significantly lower in eNpHR3-expressing mice (eNpHR3) than that in GFP-control mice (GFP). **(D)** Representative photograph of ChR2-EYFP-expressing fibers in the NAc. **(E)** Representative photograph of c-Fos expressing cells (red) in the NAc with (right) and without (left) blue light application for 1 h. **(C)** The number of c-Fos expressing cells in the NAc tended to be higher in blue light application site (on site) than that in no application site (off site). Values are means ± SEM. Data were analyzed by the Student’s *t*-test. Scale bars = 0.2 mm.

Supplementary Figure 4 Sites of bilateral Y1R agonist/antagonist injection into the NAc. Schematic drawings of the NAc from anterior to posterior according to the brain atlas (Franklin and Paxinos, 2007), illustrating the individual injection sites of Y1R agonist (circles) on the left side or Y1R antagonist (triangles) on the right side. Injections were bilateral but are only indicated on one side. Numbers indicate distance to the bregma.
