# Supplementary figures and images for: Neuropeptide Y neurons in the basolateral amygdala project to the nucleus accumbens and stimulate high-fat intake
Source: Front Cell Neurosci. 2025 Mar 27;19:1565939. doi: 10.3389/fncel.2025.1565939 (PMC11983651; doi:10.3389/fncel.2025.1565939)

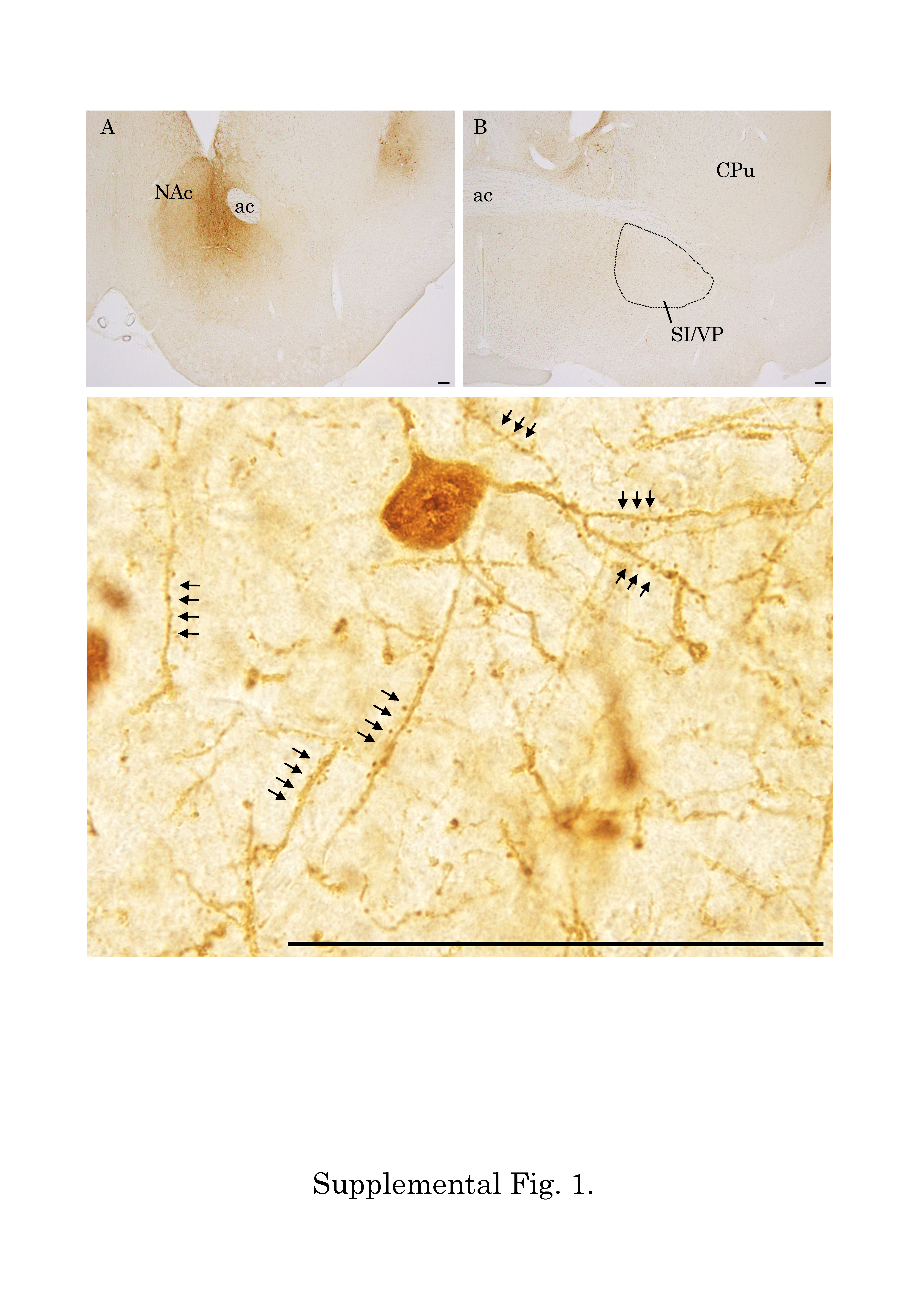

Supplement: Supplementary Figure 1 — mCherry-labeled fibers in the NAc and BLA. (A,B) Representative photograph showing the mCherry expression (brown) in the NAc (A) and substantia innominate (SI) and ventral pallidal portions of the basal forebrain (SI/VP) (B) in the AAV(retro)-FLEX-mCherry-injected mice into the NAc. (C) Representative photograph showing mCherry-positive cell and fibers in the BLA. Arrows indicate mCherry-positive dendrites bearing spines. ac; anterior commissure, CPu, caudate putamen. Scale bars = 0.1 mm. [file Image_1.jpeg]

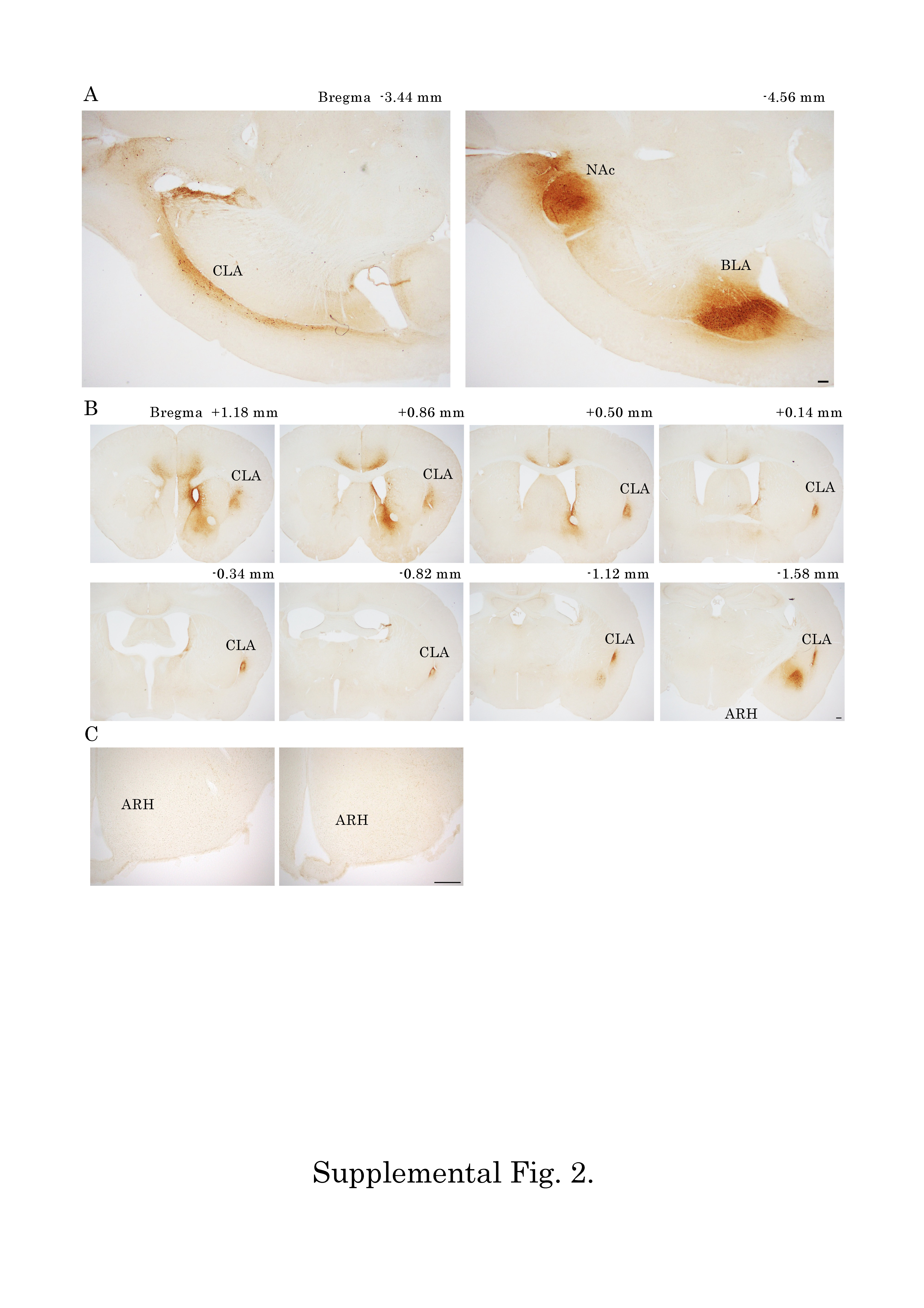

Supplement: Supplementary Figure 2 — Retrograde tracing of NPY fibers in the NAc. (A) Representative photographs of horizontal brain sections of AAV(retro)-FLEX-mCherry-NAc-injected mice at the level of the CLA (left) and NAc/BLA (right). (B) Representative coronal section showing mCherry-positive cell bodies in the CLA. Numbers indicate distance to the bregma. (C) Representative photograph of the ARH in AAV(retro)-FLEX-mCherry-NAc-injected mice. There are no mCherry-positive cells in the ARH. Scale bars = 0.2 mm. [file Image_2.jpeg]

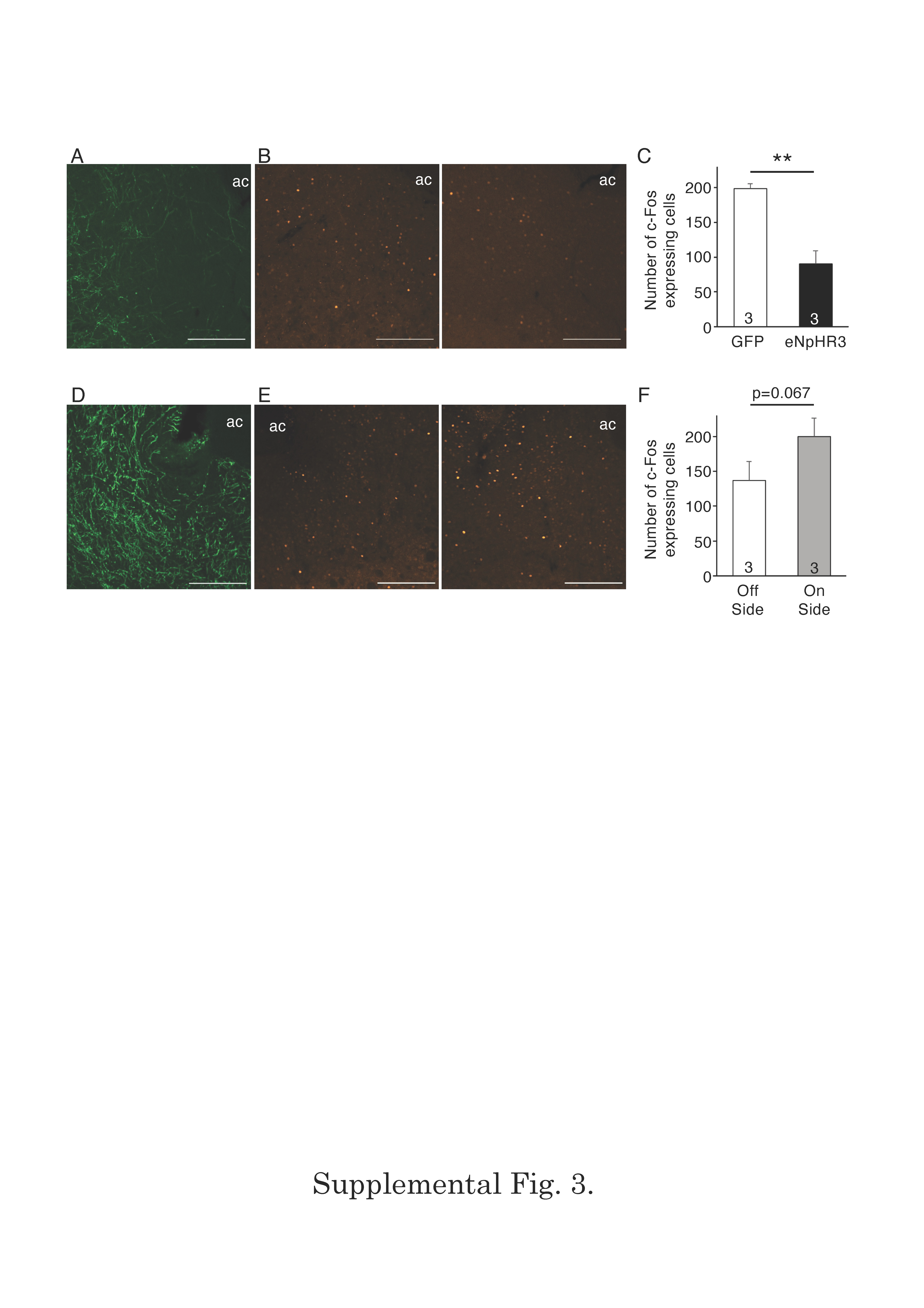

Supplement: Supplementary Figure 3 — Response of neurons in the NAc after optogenetic inhibition and stimulation of BLA-NAc NPY neurons. (A) Representative photograph of eNpHR3-EYFP-expressing fibers in the NAc. (B) Representative photograph of c-Fos expressing cells (red) in the NAc after HFD exposure with green light application for 1 h in GFP-expressing control (left) and eNpHR3-expressing mice (right). (C) The number of c-Fos expressing cells in the NAc was significantly lower in eNpHR3-expressing mice (eNpHR3) than that in GFP-control mice (GFP). (D) Representative photograph of ChR2-EYFP-expressing fibers in the NAc. (E) Representative photograph of c-Fos expressing cells (red) in the NAc with (right) and without (left) blue light application for 1 h. (C) The number of c-Fos expressing cells in the NAc tended to be higher in blue light application site (on site) than that in no application site (off site). Values are means ± SEM. Data were analyzed by the Student’s t-test. Scale bars = 0.2 mm. [file Image_3.jpeg]

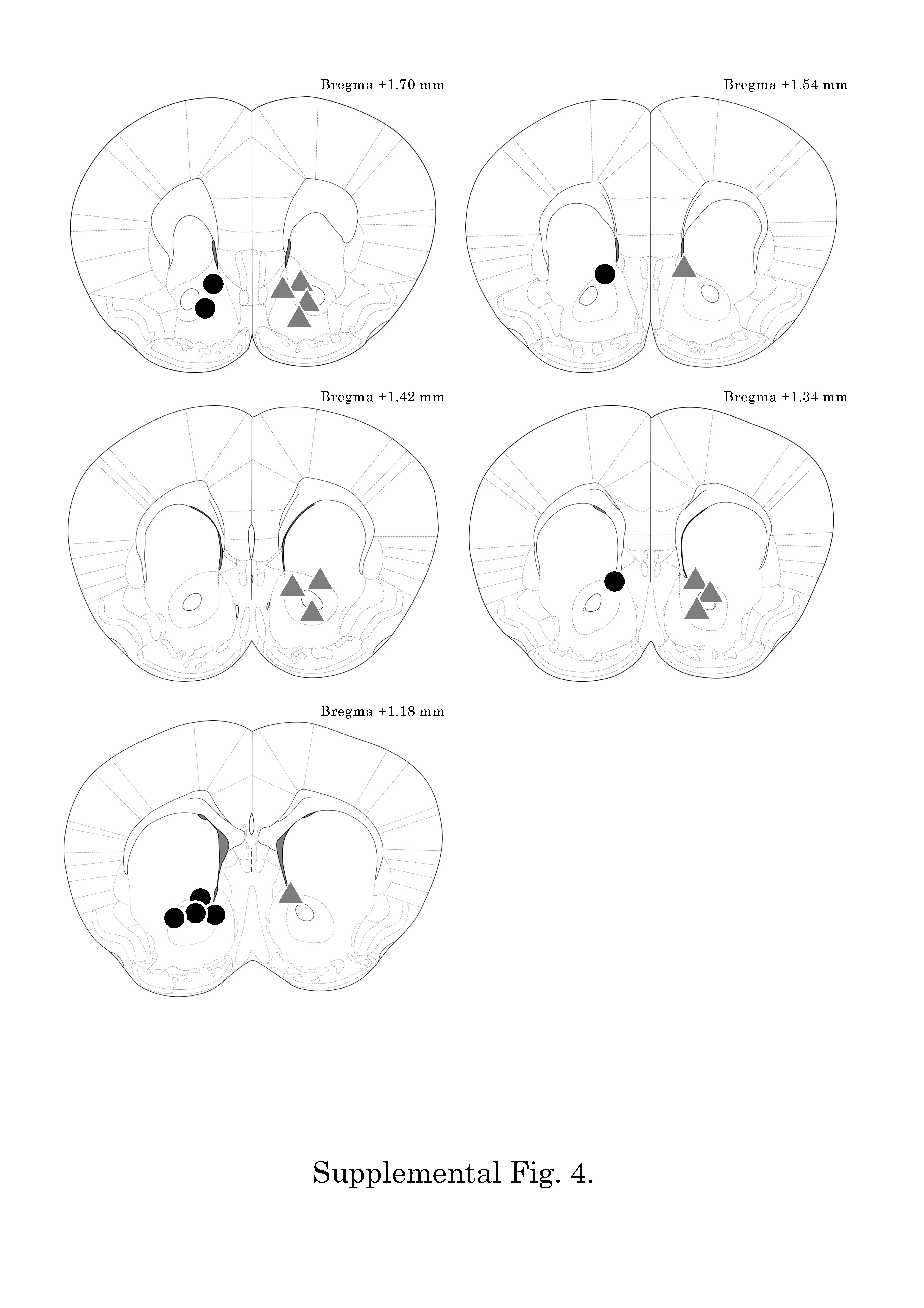

Supplement: Supplementary Figure 4 — Sites of bilateral Y1R agonist/antagonist injection into the NAc. Schematic drawings of the NAc from anterior to posterior according to the brain atlas (Franklin and Paxinos, 2007), illustrating the individual injection sites of Y1R agonist (circles) on the left side or Y1R antagonist (triangles) on the right side. Injections were bilateral but are only indicated on one side. Numbers indicate distance to the bregma. [file Image_4.jpeg]
